# The Effect of Doping on the Digital Etching of Silicon-Selective Silicon–Germanium Using Nitric Acids

**DOI:** 10.3390/nano11051209

**Published:** 2021-05-03

**Authors:** Yangyang Li, Huilong Zhu, Zhenzhen Kong, Yongkui Zhang, Xuezheng Ai, Guilei Wang, Qi Wang, Ziyi Liu, Shunshun Lu, Lu Xie, Weixing Huang, Yongbo Liu, Chen Li, Junjie Li, Hongxiao Lin, Jiale Su, Chuanbin Zeng, Henry H. Radamson

**Affiliations:** 1 Key Laboratory of Microelectronics Devices & Integrated Technology, Institute of Microelectronics, Chinese Academy of Sciences, Beijing 100029, China; liyangyang@ime.ac.cn (Y.L.); kongzhenzhen@ime.ac.cn (Z.K.); zhangyongkui@ime.ac.cn (Y.Z.); aixuezheng@ime.ac.cn (X.A.); wangguilei@ime.ac.cn (G.W.); wangqi@ime.ac.cn (Q.W.); liuziyi@ime.ac.cn (Z.L.); lushunshun@ime.ac.cn (S.L.); xielu@ime.ac.cn (L.X.); huangweixing@ime.ac.cn (W.H.); liuyongbo@ime.ac.cn (Y.L.); lichen2017@ime.ac.cn (C.L.); lijunjie@ime.ac.cn (J.L.); linhongxiao@ime.ac.cn (H.L.); sujiale@ime.ac.cn (J.S.); 2 University of Chinese Academy of Sciences, Beijing 100049, China; 3 Research and Development Center of Optoelectronic Hybrid IC, Guangdong Greater Bay Area Institute of Integrated Circuit and System, Guangzhou 510535, China; 4 Institute of Microelectronics, Chinese Academy of Sciences, Beijing 100029, China; chbzeng@ime.ac.cn

**Keywords:** vertical gate-all-around (vGAA), digital etch, quasi-atomic-layer etching (q-ALE), selective wet etching, HNO_3_ concentration, doping effect

## Abstract

Gate-all-around (GAA) field-effect transistors have been proposed as one of the most important developments for CMOS logic devices at the 3 nm technology node and beyond. Isotropic etching of silicon–germanium (SiGe) for the definition of nano-scale channels in vertical GAA CMOS and tunneling FETs has attracted more and more attention. In this work, the effect of doping on the digital etching of Si-selective SiGe with alternative nitric acids (HNO_3_) and buffered oxide etching (BOE) was investigated in detail. It was found that the HNO_3_ digital etching of SiGe was selective to n^+^-Si, p^+^-Si, and intrinsic Si. Extensive studies were performed. It turned out that the selectivity of SiGe/Si was dependent on the doped types of silicon and the HNO_3_ concentration. As a result, at 31.5% HNO_3_ concentration, the relative etched amount per cycle (REPC) and the etching selectivity of Si_0.72_Ge_0.28_ for n^+^-Si was identical to that for p^+^-Si. This is particularly important for applications of vertical GAA CMOS and tunneling FETs, which have to expose both the n^+^ and p^+^ sources/drains at the same time. In addition, the values of the REPC and selectivity were obtained. A controllable etching rate and atomically smooth surface could be achieved, which enhanced carrier mobility.

## 1. Introduction

Gate-all-around (GAA) nanowire transistors are ideal candidates for various CMOS applications due to their outstanding gate control, excellent performance, immunity to short-channel effects, and scalability [1,2,3]. Tunneling field-effect transistors (TFETs) have arisen as promising devices with emerging device concepts by breaking through the subthreshold swing limit of 60 mV/dec for low-power applications [4,5,6]. GAA nanowire TFETs have become candidates for substitutes for conventional MOS technology, especially in terms of their energy efficiency and scaling due to the better electrostatic control of the tunneling carriers provided by their nanowire structure [7,8,9,10]. SiGe channel materials have been introduced due to their excellent bandgap, high mobility, high density of states, and high compatibility with existing CMOS technology [11,12]. In order to precisely define the nanowire diameter and effective gate length, SiGe materials need to be selectively etched with accurate etching depth control and high selectivity for both n^+^-Si and p^+^-Si for CMOS and TFET applications, which have to expose both the n^+^ and p^+^ sources/drains at the same time.

Several techniques have been proposed for selective etching of SiGe, such as mixtures of HNO_3_, HF, and H_2_O [13,14,15], as well as solutions of H_2_O_2_, HF, and CH_3_COOH [16,17]. Unfortunately, the wet etching of mixtures is not appropriate for small-sized features due to the high etching rate [18,19]. Vapor etching using gaseous HCl in a chemical vapor deposition (CVD) reactor is also limited because of its high-temperature process, which degrades the sharpness of the junction [20]. Moreover, dry etching using CF_4_-based plasma has been extensively researched [21,22,23]. The disadvantage is that the plasma equipment is more complex and the loading effect is serious [24]. The etching techniques mentioned above involve continuous etching that is controlled by the etching time. Therefore, they do not meet the requirements of nano-scale transistors for process control. Atomic-layer etching (ALE) draws has significantly attracted researchers and the industrial community due to its self-limiting characteristics. The superiority of ALE techniques over other methods is due to the controllable etching rate and excellent variation control [25,26]. It has been employed for the etching of dielectrics [26,27], some nitrides [28], and metals [29,30]. Recently, an isotropic and quasi-ALE (q-ALE) method for Si-selective SiGe was proposed and reported by our group [31,32]. This q-ALE method is based on a cyclic oxidation–etching process in which hydrogen peroxide (H_2_O_2_) [32] or nitric acid (HNO_3_) [31] and buffered oxide etchants (BOEs) are separately used as an oxidant and an oxide remover agent, which is also called digital etching. The experimental etching rate of about 5 A (approximately four monolayers) per cycle accounted for the quasi-self-limited behavior in our q-ALE process [31,32]. This was explained and understood from the perspective of the activation energy, which was extracted by fitting the experimental data with the proposed oxidation model [31]. The works mentioned above mainly focused on the digital etching characteristics of SiGe that is selective of p-type doped Si. However, the digital etching of SiGe that is selective of n-type doped Si and intrinsic Si has not been studied.

In this work, the effect of doping on digital wet etching of SiGe that is selective of Si was investigated systematically. The digital etching was based on a combination of HNO_3_ and buffered oxide etchants (BOEs) as an oxidant and an oxide remover agent, respectively. The selectivity characteristics of SiGe for n^+^-Si were demonstrated. The effects of different parameters on the selectivity of the etching of germanium–silicon, such as for Si doping, HNO_3_ concentration, and SiGe doping, were examined and discussed in detail.

## 2. Materials and Methods

The substrates were 8 inch p-type Si (100) wafers with a resistivity of 8–12 ohm∙cm. The p^+^-Si/SiGe/n^+^-Si stack layers were grown in an ASM E2000 plus RPCVD reactor (ASM, Munich, Germany). First, after a standard pre-epitaxial cleaning, the wafers were baked at 900 °C in ambient H_2_ with a pressure of 20 Torr for 5 min, achieving a pure and smooth silicon surface [32]. Then, the p^+^-Si/SiGe/n^+^-Si stack layers and p^+^-Si/SiGe/i-Si stack layers were grown at 650 °C using an adjusted gas source with H_2_ as a carrier gas. Dichlorosilane (SiH_2_Cl_2_), germane (GeH_4_), diborane (B_2_H_6_), and phosphine (PH_3_) were utilized as gas precursors of Si, Ge, B, and P, respectively. The Ge incorporation, P concentration, and B concentration in silicon were achieved by tuning the gas flow and gas pressure. Finally, the epitaxial stack layers were fabricated. Then, a hard mask was deposited on the epitaxial stacked layers, and the pattern was formatted with an optical lithography with an I-line. The Si/SiGe stack layers were etched using hydrogen bromide (HBr)-based dry anisotropic etching. The details of the sample preparation can be found in [32]. Afterwards, the prepared samples were cut into same-sized slices of about 3 × 3 cm^2^ to facilitate the etching experiments.

**Figure 1:**
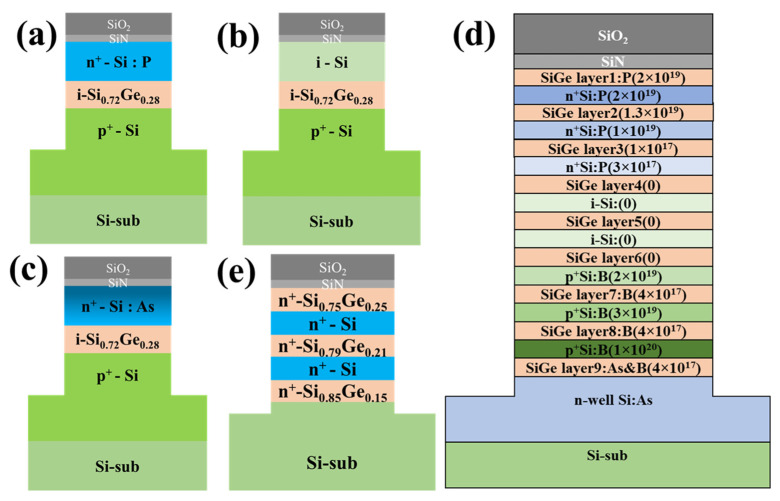
Scheme of the laminated structure with a lateral opening: (**a**) in situ n^+^-Si/i-SiGe/p^+^-Si; (**b**) i-Si/i-SiGe/p^+^-Si; (**c**) implanted n^+^-Si/i-SiGe/p^+^-Si; (**d**) SiGe/Si multilayers with different doping types; (**e**) SiGe/Si multilayers with different Ge fractions.

There were five kinds of Si/SiGe stack layer structures, as shown in Figure 1a–c. Sample I was a laminated structure in which ~300 nm p-type doped Si with a boron dopant concentration of 1.0 × 10^20^ cm^−3^, 55 nm intrinsic Si_0.72_Ge_0.28_, and 120 nm n-type doped Si with a phosphorus dopant concentration of 1.7 × 10^19^ cm^−3^ were epitaxially grown in sequence. Bottom p-type doped Si was etched to ~120 nm. The structural diagram is shown in Figure 1a. Sample II was a laminated structure in which ~300 nm p-type doped Si with a boron dopant concentration of 9 × 10^19^ cm^−3^, 55 nm intrinsic Si_0.72_Ge_0.28_, and 120 nm intrinsic Si were grown in situ and in sequence. The structural diagram is shown in Figure 1b. In Sample III, arsenic (As) ion implantation with the energy of 30 keV and dose of 4 × 10^15^ cm^−2^ was performed on the top intrinsic Si, and then 900 °C spike annealing was carried out to activate arsenic in the top Si. The structural diagram is shown in Figure 1c. This sample was employed to demonstrate the digital etching characteristics of n-type doped Si with the implantation of As. Sample IV was a laminated structure with nine Si_0.72_Ge_0.28_ layers, as shown in Figure 1d. The n^+^-SiGe layers with in situ phosphorus included SiGe layer 1, SiGe layer 2, and SiGe layer 3. The concentrations were 2 × 10^19^, 1.3 × 10^19^, and 2 × 10^19^ cm^−3^, respectively. The intrinsic SiGe layers consisted of SiGe layer 4, SiGe layer 5, and SiGe layer 6. The p^+^-SiGe layers with in situ boron included SiGe layer 7 and SiGe layer 8 with a concentration of 4 × 10^19^ cm^−3^. SiGe layer 9 was doped with boron with a concentration of 4 × 10^19^ cm^−3^ and arsenic with a concentration of 4 × 10^19^ cm^−3^. The thickness of the SiGe was ~35 nm. The thickness of the Si was ~50 nm. The sample was used for the investigation of the digital etching characteristics of doped SiGe. Sample V was a laminated structure with ~30 nm n^+^-Si layers and ~35 nm n^+^-SiGe layers with a varying Ge fraction; these layers were alternated, and the sample was used to examine the influence of the Ge mole fraction.

**Figure 2:**
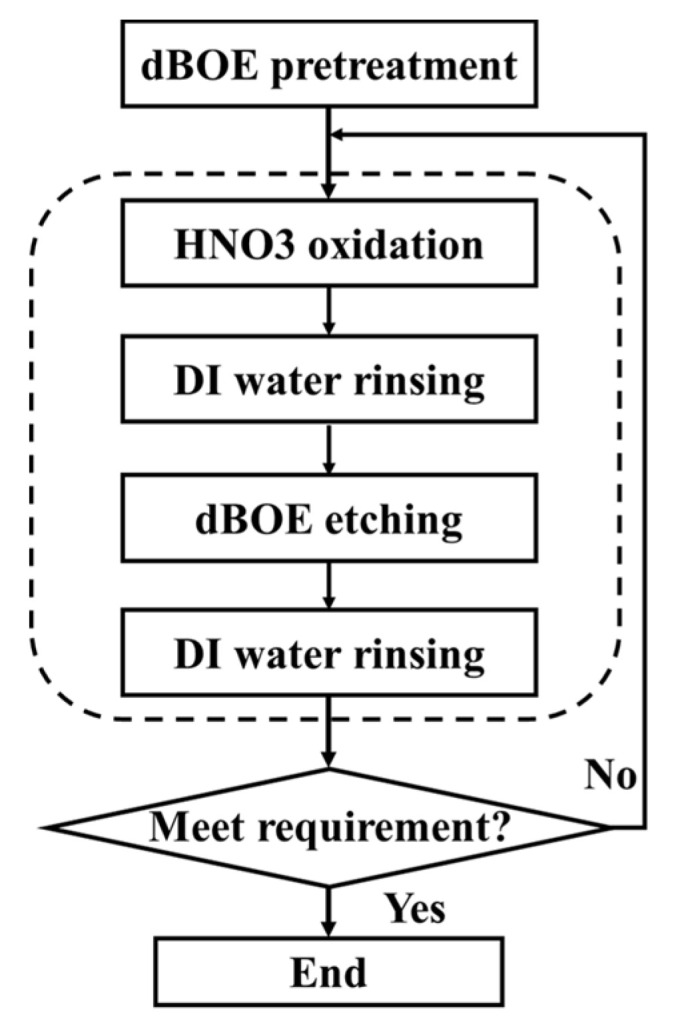
Flow diagram of the main process of digital etching.

The q-ALE process of digital wet etching, including oxidation, deionized (DI) water rinsing, oxide removal, and DI water rinsing, and the investigations on the self-limiting behavior of SiGe etching were described previously [31,32]. The flow is shown in Figure 2. The diluted BOE solutions were utilized for sample pretreatment. The steps within dotted border, including HNO_3_ oxidation, DI water rinsing, oxide removal, and DI water rinsing, were repeated for many cycles until the desired etching amount was reached. The HNO_3_ solutions in the experiments were prepared by adjusting the volume of analytical-grade nitric acid (70% (wt/wt)) and the volume of deionized water under the condition that the total volume of the solution was kept constant (2 L). The concentrations of the HNO_3_ solutions were monitored with a high-precision density meter. The values of concentrations mentioned in this paper represent mass fractions. The nitric acid solutions were employed for oxidation, and were then cooled to room temperature before use. The oxidation time was set to 60 s. It was long enough to reach saturation with an oxidation time of 27.6 s [31]. The BOE solutions used in the experiments were prepared by diluting the original BOEs (NH_4_F 34.8%, HF 6.23%) 50 times with deionized water, and the total volumes of the BOE solutions were kept at 2 L. The BOE solutions were used for oxide removal. HF/BOE concentrations that were too high would damage the Si or SiGe layers. The oxide removal time and DI water rinsing time were fixed at 60 s, which ensured the complete removal of oxides and the non-existent cross-contamination of solutions. The temperature of the control recipe was kept at room temperature (20.5 ± 0.5 °C). Unless otherwise specified, the oxidation–etching procedure in every experiment was performed for 50 cycles repeatedly. Additionally, the H_2_O_2_ (30% (wt/wt)) solutions were prepared for the H_2_O_2_ q-ALE experiments as a comparison with the HNO_3_ q-ALE experiments.

The etched morphology of the samples and the etched depth were examined with scanning electron microscopy (SEM) (Hitachi, Tokyo, Japan). Secondary ion mass spectroscopy (SIMS) was used to analyze the doping and mole fraction. Atomic force microscopy (AFM) (Dimension Icon AFM, Bruker, Billerica, MA, USA) was used to measure the surface roughness. High-resolution X-ray diffraction (HRXRD) (Delta-X, Bruker, Billerica, MA, USA) was used to determine the crystallinity and strain relaxation of the Si/SiGe/Si structures.

## 3. Results and Discussion

### 3.1. n-Type Doped Si Selectivity with H_2_O_2_ or HNO_3_ q-ALE

**Figure 3:**
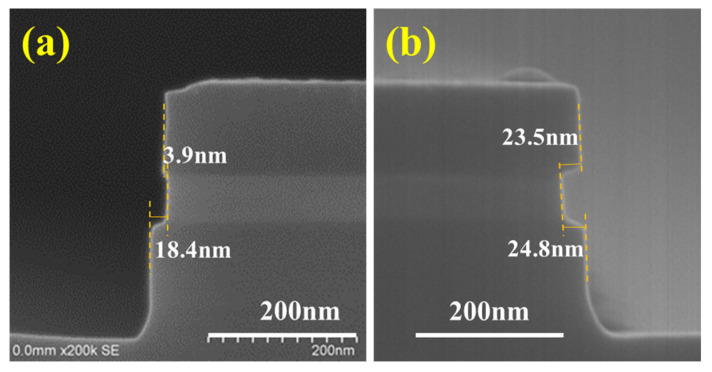
The SEM cross-section images of Sample I after digital etching at 40 cycles with (**a**) 30% H_2_O_2_-dBOE q-ALE and (**b**) 31.5% HNO_3_-dBOE q-ALE.

The digital etching of SiGe and selectivity of SiGe for p^+^-Si were previously identified with H_2_O_2_-dBOE q-ALE and HNO_3_-dBOE q-ALE [31,32]. We chose the above two q-ALE processes to investigate the selectivity of n-type silicon. Figure 3a,b show the SEM cross-section images of Sample I with n^+^-Si and in situ phosphorus after etching for 40 cycles with 30% H_2_O_2_ q-ALE and 40 cycles with 31.5% HNO_3_ q-ALE. The relative etching amounts (REAs) of SiGe/p^+^-Si with H_2_O_2_ and with HNO_3_ were 18.4 nm (see Figure 3a) and 24.8 nm (see Figure 3b), respectively. The REPC was calculated by dividing the REA by the number of etching cycles. The REPC with H_2_O_2_ was 0.46 nm, which was almost identical to the previous results [32]. The REPC with HNO_3_ was 0.62 nm, 20% higher than the previous value [31]. This may have been caused by the increase in nitric acid concentration.

**Figure 4:**
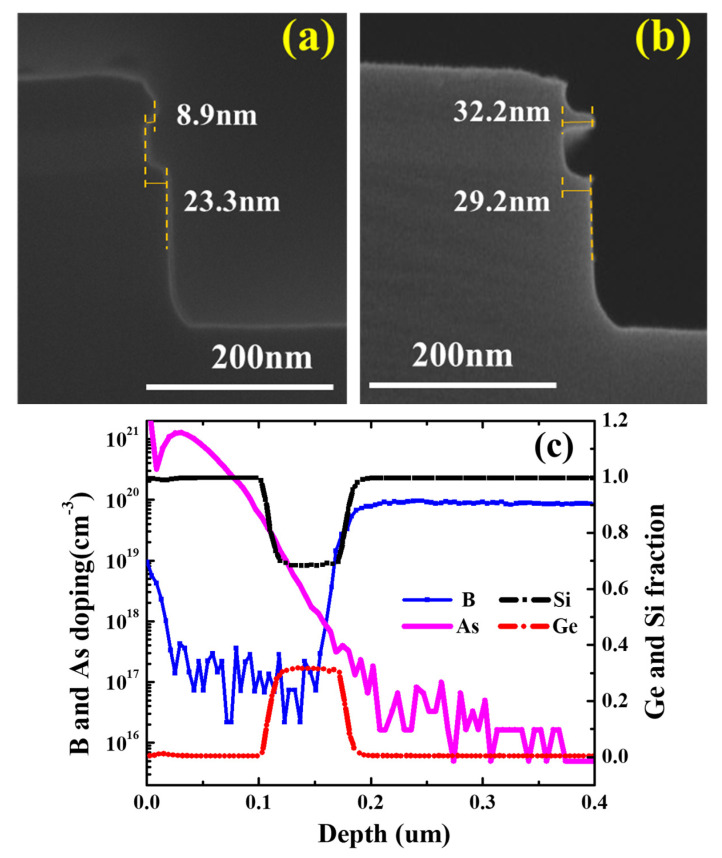
The SEM cross-section images of Sample III after digital etching at 50 cycles with (**a**) 30% H_2_O_2_-dBOE q-ALE and (**b**) 31.5% HNO_3_-dBOE q-ALE. (**c**) SIMS data of boron/arsenic and the Ge/Si mole fraction in Sample III. An abrupt B profile was formed by in situ doped epi, as the profile exhibits a large diffusion into SiGe.

It is shown in Figure 3a that the REA of SiGe/n^+^-Si with H_2_O_2_ was just 3.9 nm, which is smaller than that of SiGe/p^+^-Si, indicating poor selectivity for n^+^-Si and the high reactivity of n-Si. Therefore, H_2_O_2_-dBOE q-ALE was not suitable for p^+^-Si/SiGe/n^+^-Si structure etching. The differences between n^+^-Si and p^+^-Si in terms of selectivity and etching rate might be related to the types of carriers or the dopant types. Sample III with the arsenic ion implantation was assessed using 50 cycles with the H_2_O_2_-dBOE and HNO_3_-dBOE q-ALE process. The SEM cross-section images of Sample III are shown in Figure 4a,b. The results are almost consistent. As shown in Figure 4a, with H_2_O_2_ q-ALE, Sample III exhibited weak selectivity for n^+^-Si formed by As implantation. It was demonstrated that the carrier type—instead of dopant type—enhanced the etching rate of n^+^-Si in the H_2_O_2_ q-ALE process. The high concentration of electrons in n^+^-Si might accelerate oxide growth in H_2_O_2_ solutions, which could be explained by the improved relativity of Si-Si back bonds [33].

As shown in Figure 3a,b, the REA of SiGe/n^+^-Si with HNO_3_ was obviously larger than with H_2_O_2_, and was close to that of p^+^-Si. Similar results are shown in Figure 4a,b. This indicates the excellent selectivity for n^+^-Si with the HNO_3_ q-ALE process compared with the H_2_O_2_ q-ALE process, regardless of if in situ doped Si or implanted Si is used. In addition, the etched notch on top of Sample III shown in Figure 4b is assumed to be the result of high dose implantation. Figure 4c shows the SIMS data of boron/arsenic doping and the Ge/Si fraction. The results showed that the dopant concentration was above 1 × 10^20^ cm^−3^ within a depth of about 100 nm. Such high arsenic doping might lead to local polycrystalline or even amorphous characteristics, which enhance the etching reaction. In addition, a phenomenon that was not easy to observe was that the etching rate of SiGe near the n-type Si was slightly faster than that near the p-type Si. In the arsenic doping profile shown in Figure 4c, the arsenic was distributed in the SiGe. This might have been caused by arsenic implantations. However, the boron distribution in SiGe was negligible. It was considered that the digital etching of SiGe is dependent on the doping of SiGe. We will perform an in-depth study in the third part.

### 3.2. Effect of Doped Si and HNO_3_ Concentration Dependence

In order to explore the effect of doping in silicon on the selectivity of SiGe etching, 31.5% HNO_3_ q-ALE experiments were carried out with Sample I and Sample II. The structures of SiGe/n^+^-Si, SiGe/p^+^-Si, and SiGe/i-Si were included and could be investigated. All of the samples were processed together. Groups of samples including Sample I and Sample II were taken out every 50 cycles. For samples with different doping conditions between the top silicon and bottom silicon, the etching morphologies of the top and bottom silicon might be different. For example, the SEM images of Sample II shown in Figure S1 exhibited different REA values for SiGe/i-Si from those of SiGe/p^+^-Si and different i-Si losses with p^+^-Si loss.

**Figure 5:**
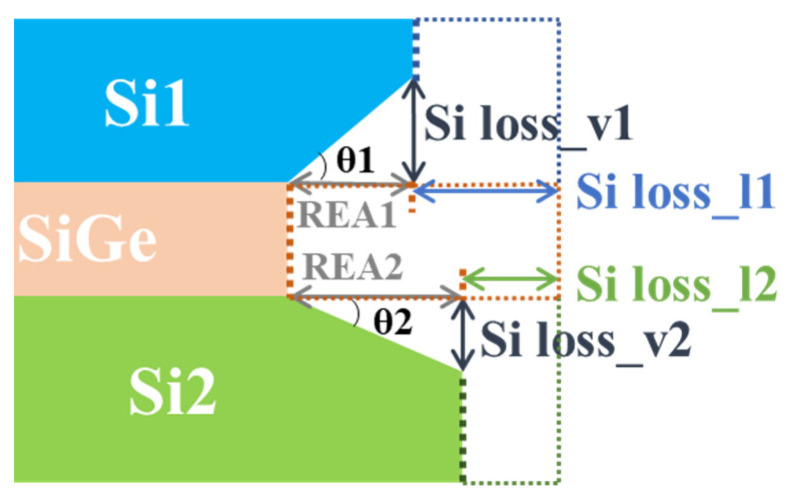
The structural diagram of the etching morphology. The dashed lines represent the initial envelope lines of the fresh sample. The solid boxes are the envelope lines as they were etched.

The structural diagram of the etching morphology is shown in Figure 5. The dashed line in Figure 5 represents the initial envelope lines of the fresh sample. The solid boxes are the envelope lines as they were etched. The angle between the etching slope at the surface of the etched Si and the horizontal direction is *θ*. Silicon was etched in the vertical and lateral directions. The etching amounts are described as the vertical Si loss (Si loss_v) and lateral Si loss (Si loss_l). As discussed in a previous work [31], the influence of crystal planes on the etching rate was ignored, that is, Si loss_v was almost equal to Si loss_l. The etching amount in the vertical direction could be directly measured. Therefore, the etching amount in the vertical direction is usually regarded as the Si etching amount (Si loss) in the following section. Silicon–germanium was only etched laterally. The etching amount can be described as the sum of the REA and Si loss_l. The selectivity can be expressed as the ratio of SiGe loss to Si loss, as described in Equation (1).

(1) $$selectivity=\frac{\left(REA+Si\;loss\right)}{Si\;loss}=1+cot\theta$$

In addition, in Sample I and Sample II, the diffusion of impurities from silicon to silicon–germanium was negligible, and SiGe could be regarded as intrinsic. The germanium component was fixed in the whole SiGe layer. Therefore, the q-ALE etching of SiGe/Si1 and the q-ALE etching of SiGe/Si2 were independent of each other. The selectivity could be separately calculated by using the Equation (1). According to the angle calculation method and the length calculation method, the values of the selection ratios were very close. This was proved by our experimental data.

**Figure 6:**
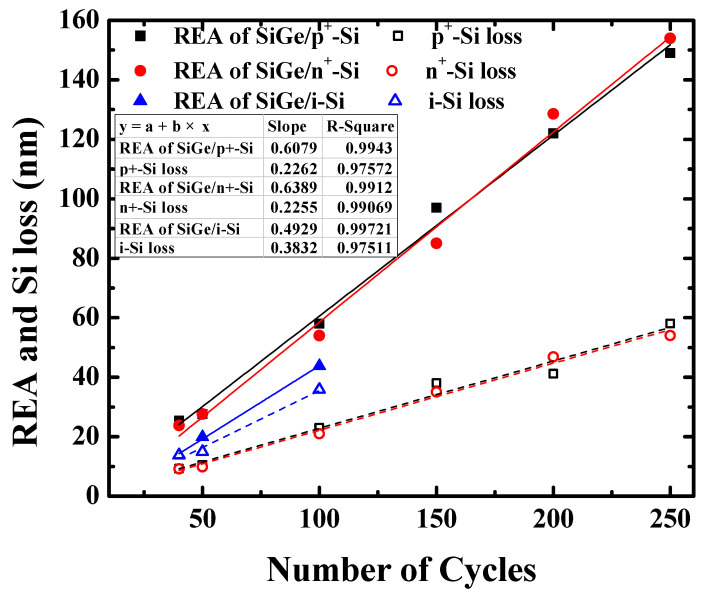
Dependence of the REA and Si loss on the number of etching cycles for SiGe/n^+^-Si, SiGe/p^+^-Si, and SiGe/i-Si. The scatters are the experimental data, and the lines are the linear fitting curves of the experimental data. The slopes represent the REPC and silicon etching amounts for each cycle (EPC).

Figure 6 shows the dependence of the REA and Si loss on the number of etching cycles for SiGe/n^+^-Si, SiGe/p^+^-Si, and SiGe/i-Si. The scatters in Figure 6 are the data points obtained through the experiments, and the lines are the curves fitted linearly according to the experimental data. It is shown that the REA of SiGe/n^+^-Si, REA of SiGe/p^+^-Si, and REA of SiGe/i-Si were highly linear dependent on the number of etching cycles, which was confirmed by the R_square up to 0.975. The Si losses of n^+^-Si, p^+^-Si, and i-Si were also linearly related to the number of cycles. In the table embedded in Figure 6, the fitting slopes of the REA curves and the Si loss curves represent the REPC and silicon etching amount of each cycle (EPC). It was shown that the REPC of SiGe/p^+^-Si was 0.6079 nm, which was close to the REPC value of SiGe/n^+^-Si (0.6389 nm). The EPC of p^+^-Si was 0.2262 nm, which was also close to the EPC value of n^+^-Si (0.2255 nm). The results indicate that the 31.5% HNO_3_ concentration had the same etching rate for p^+^-Si and n^+^-Si. The concentration is expected to be used for the digital etching of p^+^-Si/SiGe/n^+^-Si stack structures, such as GAA CMOS and TFET applications. Moreover, the slopes in the fitting curves of SiGe/i-Si REA are lower than that of doped Si, suggesting its poor selectivity for i-Si. The EPC of i-Si was 0.3732 nm. It was demonstrated that the etching rate was larger than that of doped Si. It was considered that the doping of silicon contributed to the better selectivity for silicon with the nitric acid etching of SiGe.

**Figure 7:**
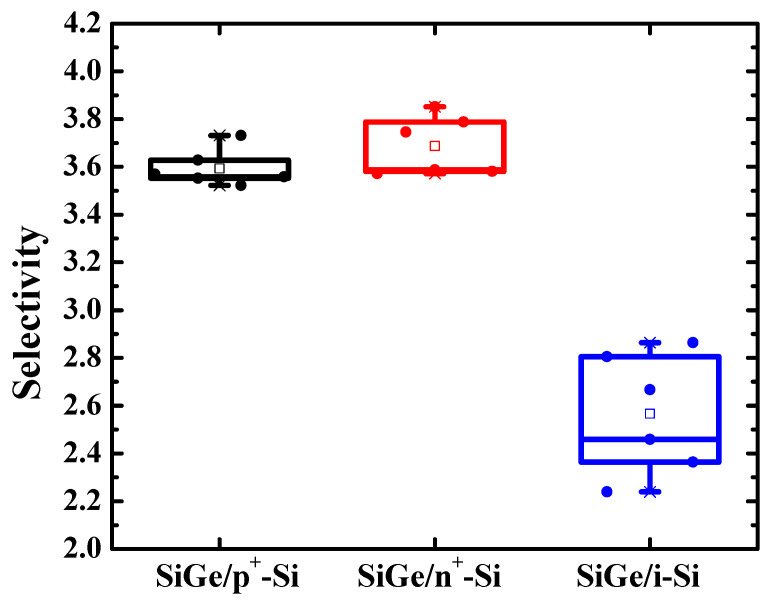
Box plot of the selectivity of SiGe/n^+^-Si, SiGe/p^+^-Si, and SiGe/i-Si. The means and the standard deviations are 3.59, 3.68, and 2.56 and 0.0759, 0.1228, and 0.2512, respectively. Significant improvements in the selectivity for doped Si were observed. The selectivity of SiGe/n^+^-Si and SiGe/p^+^-Si was similar, but the variation in SiGe/n^+^-Si was larger.

Figure 7 shows the selectivity of SiGe/n^+^-Si, SiGe/p^+^-Si, and SiGe/i-Si. The selectivity was calculated with Equation (1). The experimental data were obtained by measuring the SEM images of Sample I and Sample II with the 31.5% HNO_3_ q-ALE process. We carried out the experiments six times on Sample I and Sample II, and six sets of data were obtained. The mean values of the SiGe/n^+^-Si, SiGe/p^+^-Si, and SiGe/i-Si selectivity were 3.59, 3.68, and 2.56, respectively. The values of the standard deviations were 0.0759, 0.1228, and 0.2512, respectively. The results show the significant improvements in selectivity for doped Si compared with intrinsic Si. The selectivity of SiGe/n^+^-Si and SiGe/p^+^-Si was similar—40% larger than that of intrinsic silicon. It was demonstrated that doped Si was more difficult to etch in the process of digital etching, which might be due to the oxidation difficulty in the HNO_3_ solutions. Moreover, it was observed that the variation in SiGe/n^+^-Si was larger than that in SiGe/p^+^-Si. This indicates that it is more susceptible to process factors, such as concentration monitoring and solution preparation. The selectivity might be sensitive to the actual HNO_3_ concentration.

**Figure 8:**
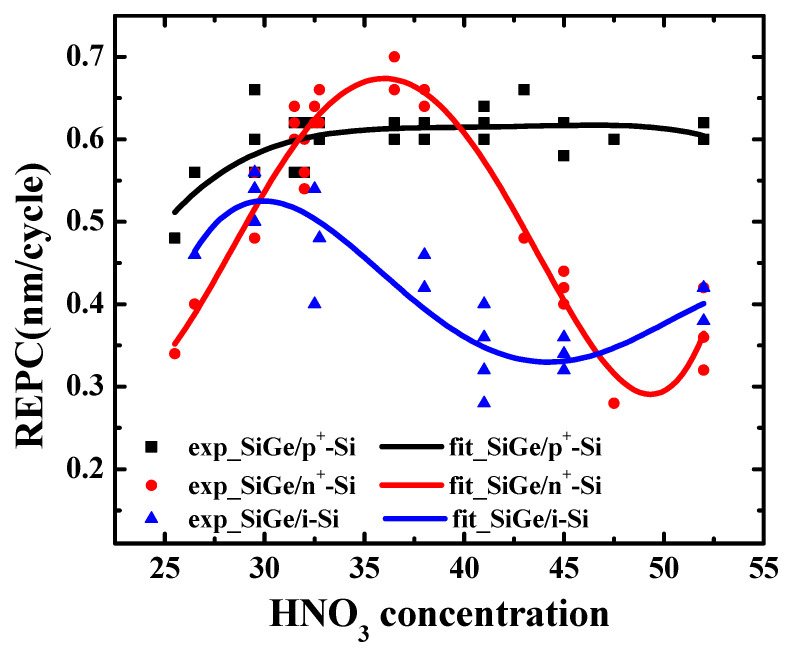
REPC of SiGe/n^+^-Si, SiGe/p^+^-Si, and SiGe/i-Si as a function of HNO_3_ concentration. The dots in the figure are the experimental data, and the lines are the fitting curves of the experimental data. The slopes represent the relative etching amount per cycle (REPC) and the etching amount per cycle (EPC) of silicon.

In order to explore the effect of nitric acid concentration, we carried out digital etching experiments on Sample I and Sample II with different HNO_3_ concentrations. Figure 8 shows the REPC of SiGe/n^+^-Si, SiGe/p^+^-Si, and SiGe/i-Si as a function of HNO_3_ concentration. As described in Figure 8, with the increase in HNO_3_ concentration, the REPC of SiGe/p^+^-Si increased and became saturated at 0.61 nm/cycle with the 29.5% HNO_3_ concentration. The appearance of saturation is helpful for the stability of the process. However, to achieve accurate etching control for small-sized devices, a controllable etching rate is expected. Moreover, when the concentration was lower than 26.5%, the etched surface of SiGe was very rough, as shown in (Supporting Information, Figure S2a). In the case of high concentrations, damage occurred on the etched surface, as shown in (Supporting Information, Figure S2c). The critical concentration might be between 47.5% and 52%, and the 52% concentration exceeded this limit, resulting in etching damage as shown in (Supporting Information, Figure S2c,d). Therefore, there is a tradeoff between the controllable etching rate, high etching control, small process variations, and excellent etched surfaces when choosing a HNO_3_ concentration for the digital etching of SiGe that is selective of p^+^-Si.

As discussed above, the concentration range from 26.5% to 47.5% led to an etching morphology with a smooth surface that was free of damage, showing that the study on HNO_3_ concentration was meaningful. In this concentration range, it was observed that the REPC of SiGe/n^+^-Si had a trend of first increasing and then decreasing. At the concentration of 36.5%, the REPC reached the maximum. The etching rate might be the least influenced by concentration fluctuations. The HNO_3_ concentration might be used for the fabrication of GAA transistors due to the small process variations. At the concentrations of 31.5% and 40%, the fitting curves of SiGe/n^+^-Si and SiGe/p^+^-Si intersected. Despite its identical relative etching rate, the 40% HNO_3_ concentration requires a greater nitric acid concentration, thus increasing the cost. It was considered that 31.5% is the most suitable concentration for the digital etching of p^+^-Si/SiGe/n^+^-Si stack structures, such as for GAA CMOS and TFET applications, which must expose both the n^+^ and p^+^ sources/drains at the same time.

For the digital etching of SiGe/i-Si, the REPC first increased and then decreased with the increase in HNO_3_ concentration. The REPC of SiGe/i-Si reached the maximum, which was equal to that of SiGe/n^+^-Si at the 30% HNO_3_ concentration. However, there was a large process variation, which is a burden in the control of the etching process. Through many repeated experiments with fine concentration intervals, the HNO_3_ concentration relationship can be further verified.

### 3.3. Effect of Doped SiGe and Ge Fraction Dependence

**Figure 9:**
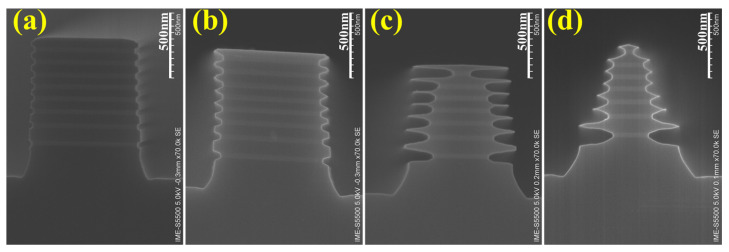
The SEM cross–section images of Sample IV after digital etching with 31.5% HNO_3_-dBOE q-ALE at (**a**) 40 cycles, (**b**) 100 cycles, (**c**) 200 cycles, and (**d**) 300 cycles.

In the first part, it was observed that the diffusion of arsenic into SiGe might enhance the etching rate of SiGe. Sample IV with in situ doping in SiGe was treated with the 31.5% HNO_3_ q-ALE process. To make the effect of doping more obvious and easier to observe, 300 cycles of etching were performed. Figure 9 shows the SEM cross-section images of Sample IV after digital etching at 40, 100, 200, and 300 cycles with 31.5% HNO_3_-dBOE q-ALE. It was shown that SiGe layer 1 disappeared at 100 cycles, which might have been due to the etching from the top. The SiGe layer 2 was penetrated horizontally at 200 cycles. It was observed that the remaining SiGe layer 3 at 300 cycles was the lowest. The etching amounts of the intrinsic SiGe layers, including SiGe layer 4, SiGe layer 5, and SiGe layer 6, were almost equal—slightly more than in the p-type SiGe, such as in SiGe layer 7 and SiGe layer 8. The results demonstrate that the relationship of the etching rate with the doping type is: p-type < intrinsic < n-type. The etching rate increased with the increase in n-type dopant concentration. As shown in Figure 9, the remaining amount of SiGe layer 9 doped by almost equal concentrations of arsenic and boron was similar to that of the intrinsic SiGe layers. This indicates the dependence on the carrier type instead of the dopant type. Additionally, it is demonstrated that the etching selectivity between the same doped SiGe and Si always exists regardless of the doping type.

**Figure 10:**
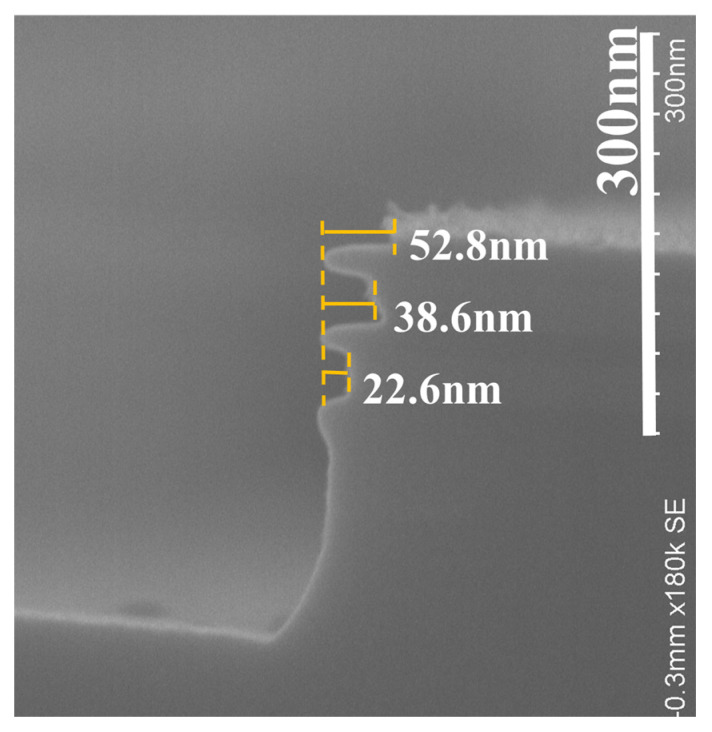
The SEM cross–section images of Sample V after digital etching with 31.5% HNO_3_-dBOE q-ALE for 100 cycles.

To investigate the influence of the Ge fraction on the selectivity and etching rate of n^+^-SiGe/n^+^-Si, Sample V was etched with 31.5% HNO_3_-dBOE q-ALE for 100 cycles. Figure 10 shows the SEM cross-section images of Sample V after digital etching with 31.5% HNO_3_-dBOE q-ALE for 100 cycles. As shown in Figure 10, there is a selectivity for n^+^-Si in the n^+^-SiGe digital etching. The top SiGe might have been etched from the top opening. The REA of the n^+^-SiGe increased with the increase in the Ge fraction. This might have been due to the easier hole injection and larger valence band offset [18]. It was demonstrated that increasing the Ge fraction could increase the etching rate of n^+^-SiGe and the selectivity of n^+^-SiGe/n^+^-Si.

### 3.4. Strain and Material Quality Analyses

**Figure 11:**
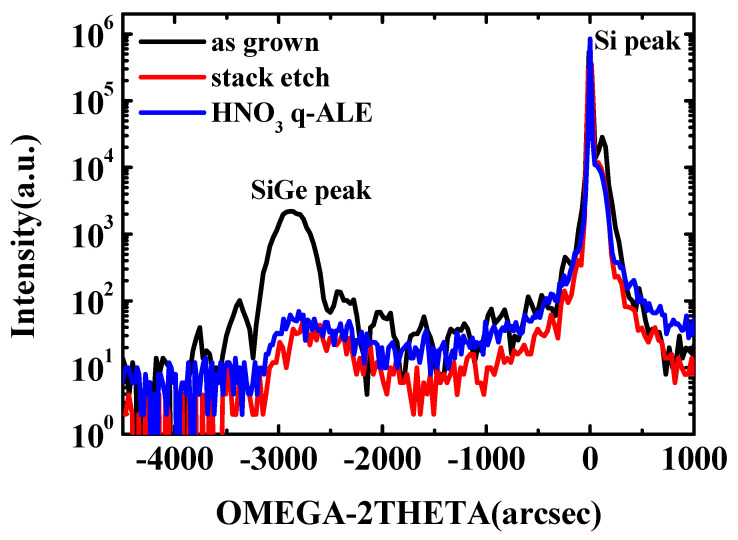
HRXRD rocking curves around the (004) reflection of the as–grown p^+^-Si/SiGe/n^+^-Si stack layers after vertical stack etching and after 31.5% HNO_3_ q-ALE with 50 cycles.

In order to further determine the strain and material quality of the samples after the etching process, the HRXRD analysis scanning around the (004) diffraction order has been performed on p^+^-Si/SiGe/n^+^-Si stack layers as grown, after vertical stack etch, and after SiGe q-ALE with 31.5% HNO_3_. The HRXRD rocking curves are shown in Figure 11. For the epitaxial growth sample, the SiGe signal was intense, and many fringes were observed around the SiGe peak due to X-ray interference at the SiGe/Si interface, which indicated a high-quality SiGe/Si interface. Therefore, the subsequent etching experiments could be implemented based on the high-quality epitaxial film.

A high full-width at half-maximum (FWHM) is a characteristic of a material’s quality [34]. Compared with the epitaxial growth sample, the intensity of the SiGe peak after the etching process was weaker, which might be due to the reduction of SiGe material into chips after etching. There was also a slight shift of the SiGe peak towards the Si peak, which is an indicator of strain in the SiGe layer. As shown, the SiGe peak of the stack-etched sample was shifted toward the Si substrate peak compared to the SiGe peak of the as-grown sample. This was a result of a strain relaxation induced by the stack-etching process. No continued shift of SiGe peak was detected after SiGe q-ALE etching, indicating that there was no further strain relaxation. This is important point out in the SiGe channel because the energy band and carrier mobility are dependent on the strain.

**Figure 12:**
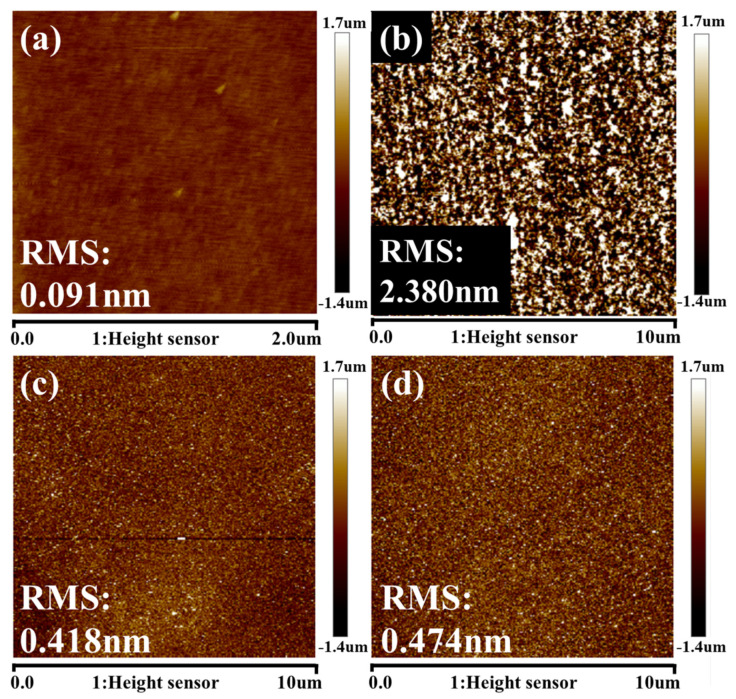
Typical AFM images of flat (100) Si_0.72_Ge_0.28_ surfaces before and after the etching process: (**a**) as–grown; (**b**) HNO_3_:HF:H_2_O mixtures; (**c**) q-ALE with 50 cycles; (**d**) q-ALE with 30 cycles.

In vertical GAA CMOS and TFET applications, SiGe is often used as a channel material, and the etched surface can be used as a channel interface. It is necessary to check the surface roughness after it is etched. Figure 12 shows the AFM morphology of the SiGe surface on as-grown epi-SiGe after etched with HNO_3_:HF:H_2_O mixtures and after etched with q-ALE. It was found that the root mean square (RMS) roughness of the q-ALE process still maintained a comparatively low value after many cycles. The RMS was 0.418 nm at 50 cycles and 0.474 nm at 30 cycles. AFM measurements were performed at many sites. The RMS was always in the range of 0.40 to 0.50 nm. It turned out that the RMS variation was due to differences in the test sites, and there was no dependence on the number of cycles. It was demonstrated that the surface roughness after the HNO_3_-dBOE q-ALE process stayed in the range of 0.40 to 0.50 nm and was better than dry [35] and wet chemical continuous etching.

## 4. Conclusions

The HNO_3_-dBOE q-ALE process consists of alternative HNO_3_ oxidation and dBOE oxide removal. Compared with the H_2_O_2_-dBOE q-ALE process, excellent selectivity for n-type doped Si could be found with HNO_3_-dBOE q-ALE. Doping plays an important role in the selective etching of SiGe. The selectivity of SiGe/Si was enhanced by doped Si. In addition, the selectivity for n-type doped Si had a strong dependence on the HNO_3_ concentration. The relative etching of n^+^-Si reached a maximum at 36.5% HNO_3_ concentration, and p^+^-Si was saturated at 29.5% HNO_3_ concentration. It was found that at 31.5% HNO_3_ concentration, identical selectivity levels for p^+^-Si and n^+^-Si could be achieved. The REPC was 0.6 nm. The etching selectivity was 3.6–40% higher than that of intrinsic Si. The most suitable concentration for digital etching of p^+^-Si/SiGe/n^+^-Si stack structures, such as for GAA CMOS and TFET applications, which have to expose both the n^+^ and p^+^ sources/drains at the same time, is considered to be 31.5%. The relationship between the etching rate of doped SiGe and the doping type is: p-type < intrinsic < n-type. The etching rate of doped SiGe could be improved by the Ge fraction. Finally, this technique is a promising process for the fabrication of GAA CMOS transistors and TFETs due to its perfectly controllable etching rate and the resulting atomically smooth surface roughness.

## Data Availability

The data presented in this study are available on request from the corresponding author.

## Supplementary Materials

The following are available online at https://www.mdpi.com/article/10.3390/nano11051209/s1, Figure S1: The SEM cross-section images of Sample II after digital etching at 50 cycles: (a) in 30% H_2_O_2_-dBOE q-ALE (b) 31.5% HNO_3_-dBOE q-ALE, Figure S2: The SEM cross–section images of Sample I after HNO_3_ digital etching at 50 cycles with varying HNO_3_ concentrations: (a) 25.5% HNO_3_ concentration (b) 36.5% HNO_3_ concentration (c) 52% HNO_3_ concentration, the etch damage is marked in the yellow dotted line. (d) significant etch damage at 52% HNO_3_ concentration.
