# Supplementary material for: The Effect of Doping on the Digital Etching of Silicon-Selective Silicon–Germanium Using Nitric Acids
Source: Nanomaterials (Basel). 2021 May 3;11(5):1209. doi: 10.3390/nano11051209 (PMC8147633; doi:10.3390/nano11051209)
Supplement: Supplementary file 1 [file nanomaterials-11-01209-s001.zip › nanomaterials-1171979-SI.pdf]

nanomaterials

Supporting Information

The Effect of Doping on the Digital Etching of Silicon-Selective Silicon–Germanium Using Nitric Acids

Yangyang Li [1,2], Huilong Zhu [1,*], Zhenzhen Kong [1], Yongkui Zhang [1], Xuezheng Ai [1], Guilei Wang [1,2,3], Qi Wang [1], Ziyi Liu [1,2], Shunshun Lu [1], Lu Xie [1,2], Weixing Huang [1,2], Yongbo Liu [1,2], Chen Li [1,2], Junjie Li [1], Hongxiao Lin [1,3], Jiale Su [1], Chuanbin Zeng [4] and Henry H. Radamson [1,2,3,*]

[1] Key Laboratory of Microelectronics Devices & Integrated Technology, Institute of Microelectronics,
Chinese Academy of Sciences, Beijing 100029, China; liyangyang@ime.ac.cn (Y.L.); kongzhenzhen@ime.ac.cn (Z.K.); zhangyongkui@ime.ac.cn (Y.Z.); aixuezheng@ime.ac.cn (X.A.); wangguilei@ime.ac.cn (G.W.); wangqi@ime.ac.cn (Q.W.); liuziyi@ime.ac.cn (Z.L.); lushunshun@ime.ac.cn (S.L.); xielu@ime.ac.cn (L.X.); huangweixing@ime.ac.cn (W.H.); liuyongbo@ime.ac.cn (Y.L.); lichen2017@ime.ac.cn (C.L.); lijunjie@ime.ac.cn (J.L.); linhongxiao@ime.ac.cn (H.L.), sujiale@ime.ac.cn (J.S.)
[2] University of Chinese Academy of Sciences, Beijing 100049, China
[3] Research and Development Center of Optoelectronic Hybrid IC, Guangdong Greater Bay Area Institute of Integrated Circuit and System, Guangdong 510535, China
[4] Institute of Microelectronics, Chinese Academy of Sciences, Beijing 100029, China; chbzeng@ime.ac.cn
* Correspondence: zhuhuilong@ime.ac.cn (H.Z.); rad@ime.ac.cn (H.H.R.)

1. *intrinsic Si selectivty with H_2O_2 or HNO_3 q-ALE*

Figure S1a and S1b show the SEM cross–section images of Sample II with the i-Si/i-SiGe/p$^+$-Si laminated structure after etching with 30% H_2O_2 q-ALE and 31.5% HNO_3 q-ALE 50 cycles, respectively. The relative etch amount (REA) of SiGe/p$^+$-Si with H_2O_2 and with HNO_3 is 24.8 nm (see Figure S1a and 30.4 nm (see Figure S1b) respectively. The REPC with H_2O_2 is identical to the previous data. The REPC with HNO_3 is ~0.6 nm, higher than the previous value due to the increase of nitric acid concentration. It is shown in Figure S1a that the REA values of SiGe/i-Si with H_2O_2 and HNO_3 are identical, ~14 nm, smaller than that of SiGe/p$^+$-Si, indicating poor selectivity to i-Si. This might be due to high reactivity of i-Si. It is illustrated that the selectivity of the digital etch SiGe to Si could be enhanced by doped Si.

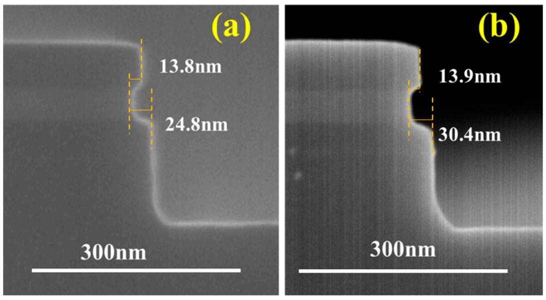

Figure S1. The SEM cross–section images of Sample II after digital etching at 50 cycles: (**a**) in 30% H_2O_2-dBOE q-ALE (b) 31.5% HNO_3-dBOE q-ALE.

2. *Dependence etch surface on HNO_3 concentration*

Figure S2 shows the SEM cross–section images of Sample I with the n^+-Si/i-SiGe/p^+-Si laminated structure after HNO_3 q-ALE etching 50 cycles with varying HNO_3 concentrations. As shown, the relative etch amount (REA) of SiGe/n^+-Si increases first and then decreases with the increase of HNO_3 concentration. It is demonstrated that the selectivity of n^+-Si is dependent on HNO_3 concentration.

It is shown in Figure S2a that the etched surface of SiGe is very rough at 25.5% HNO_3 concentration, indicating difficulty in etch reactivity. And, at 52% HNO_3 concentration, the rough surface might be due to etch damage. The significant etch damage could be observed in Figure S2d.

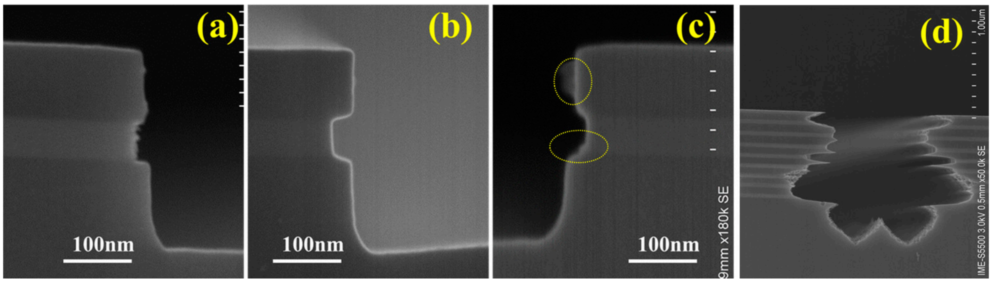

Figure S2. The SEM cross–section images of Sample I after HNO_3 digital etching at 50 cycles with varying HNO_3 concentrations: (**a**) 25.5% HNO_3 concentration (b) 36.5% HNO_3 concentration (c) 52% HNO_3 concentration, the etch damage is marked in the yellow dotted line. (d) significant etch damage at 52% HNO_3 concentration.
